# C–C chemokine receptor 5 is essential for conventional NK cell trafficking and liver injury in a murine hepatitis virus-induced fulminant hepatic failure model

**DOI:** 10.1186/s12967-023-04665-8

**Published:** 2023-11-29

**Authors:** Yun-Hui Liu, Lin Zhu, Zhong-Wei Zhang, Ting-Ting Liu, Qiu-Yu Cheng, Meng Zhang, Yu-Xin Niu, Lin Ding, Wei-Ming Yan, Xiao-Ping Luo, Qin Ning, Tao Chen

**Affiliations:** 1 Department of Infectious Diseases, Tongji Hospital, Tongji Medical College and State Key Laboratory for Diagnosis and Treatment of Severe Zoonostic Infectious Disease, Huazhong University of Science and Technology, 1095, Jiefang Avenue, Wuhan, 430030 People’s Republic of China; 2 National Medical Center for Major Public Health Events, Wuhan, 430030 Hubei Province China; 3 Department of Pediatrics, Tongji Hospital, Tongji Medical College, Huazhong University of Science and Technology, 1095, Jiefang Avenue, Wuhan, 430030 Hubei Province China

**Keywords:** Virus infection, Liver failure, NK cell, Chemotaxis, CCR5

## Abstract

**Background:**

Previous studies have demonstrated that natural killer (NK) cells migrated into the liver from peripheral organs and exerted cytotoxic effects on hepatocytes in virus-induced liver failure.

**Aim:**

This study aimed to investigate the potential therapeutic role of chemokine receptors in the migration of NK cells in a murine hepatitis  virus strain 3 (MHV-3)-induced fulminant hepatic failure (MHV-3-FHF) model and its mechanism.

**Results:**

By gene array analysis, chemokine (C–C motif) receptor 5 (CCR5) was found to have remarkably elevated expression levels in hepatic NK cells after MHV-3 infection. The number of hepatic CCR5^+^ conventional NK (cNK) cells increased and peaked at 48 h after MHV-3 infection, while the number of hepatic resident NK (rNK) cells steadily declined. Moreover, the expression of CCR5-related chemokines, including macrophage inflammatory protein (MIP)-1α, MIP-1β and regulated on activation, normal T-cell expressed and secreted (RANTES) was significantly upregulated in MHV-3-infected hepatocytes. In an in vitro Transwell migration assay, CCR5-blocked splenic cNK cells showed decreased migration towards MHV-3-infected hepatocytes, and inhibition of MIP-1β or RANTES but not MIP-1α decreased cNK cell migration. Moreover, CCR5 knockout (KO) mice displayed reduced infiltration of hepatic cNK cells after MHV-3 infection, accompanied by attenuated liver injury and improved mouse survival time. Adoptive transfer of cNK cells from wild-type mice into CCR5 KO mice resulted in the abundant accumulation of hepatic cNK cells and aggravated liver injury. Moreover, pharmacological inhibition of CCR5 by maraviroc reduced cNK cell infiltration in the liver and liver injury in the MHV-3-FHF model.

**Conclusion:**

The CCR5-MIP-1β/RANTES axis played a critical role in the recruitment of cNK cells to the liver during MHV-3-induced liver injury. Targeted inhibition of CCR5 provides a therapeutic approach to ameliorate liver damage during virus-induced acute liver injury.

**Supplementary Information:**

The online version contains supplementary material available at 10.1186/s12967-023-04665-8.

## Introduction

Fulminant hepatic failure (FHF) is a serious challenge in clinical practice and is characterized by acute and severe hepatic damage and dysfunction, combined extrahepatic organ injuries, and high short-term mortality. Of the various causes of FHF, hepadnaviruses were the major cause in developing countries, while alcohol and autoimmune hepatitis were the most common causes of FHF in developed countries [1, 2]. Although systemic treatments, including antiviral treatment, hepatocyte regeneration, and artificial extracorporeal liver support, have been established for clinical practice, specific and effective intervention targets are still absent due to the lack of a full pathophysiological understanding of the disease [2, 3].

Immune activation-associated liver injury has been proven to be one of the crucial mechanisms of hepatitis virus-induced liver failure [4, 5]. Natural killer (NK) cells function as the primary line of defence against pathogens after viral infection [6]. Previous studies have demonstrated that two subsets of hepatic NK cells, conventional NK (cNK) and liver resident NK (rNK) cells, play important functional roles during virus invasion. The two populations of NK cells were distinguished by the expression of CD49a and CD49b, as CD49a^−^CD49b^+^ and CD49a^+^CD49b^–^ cells for cNK and rNK cells, respectively [7]. Our previous research revealed that NK cells might play a hepatic cytotoxic role after migrating to the liver from peripheral organs during murine hepatitis virus strain-3 (MHV-3) infection [8]. Nevertheless, the underlying mechanism of NK cell subset migration into the liver remains poorly defined.

Chemokine (C–C motif) receptor 5 (CCR5) is a member of chemokine receptors, a subclass of the GPCR (G-protein-coupled receptor) superfamily. It is expressed on diverse immunocytes, including NK cells, NKT cells, CD4^+^ T cells and macrophages [9–12], and interacts with chemokines during immune chemotaxis. CCR5 is responsible for regulating the immune reaction by interacting with CCR5-corresponding chemokines, including macrophage inflammatory protein (MIP)-1α, MIP-1β and regulated on activation, normal T-cell expressed and secreted protein (RANTES), which are also called chemokine (C–C motif) ligand 3 (CCL-3), CCL-4 and CCL-5, respectively [13]. Previous studies have suggested that CCR5 is crucial to the progression of liver diseases [14–18]. Although increased hepatic expression of CCR5-corresponding chemokines was observed in biopsies from patients with FHF [19, 20], the detailed contribution of CCR5 ligands or CCR5 to the process of liver damage remains unknown.

In our present research, we demonstrated that CCR5 played a prominent role in cNK cell migration to the liver, leading to severe liver damage during MHV-3-FHF. Moreover, pharmacological inhibition of cNK cell infiltration by blocking CCR5 could attenuate liver damage and prolong the survival time of mice, which provides a therapeutic approach to alleviate liver impairment during virus-induced liver injury.

## Materials and methods

### Animals and treatments

Wild-type (WT) BALB/cJ mice were purchased from Vital River Laboratory Animal Technology (Beijing, China). CCR5 KO mice on a BALB/cJ background were constructed by GemPharmatech LLC (Nanjing, China) and showed no overt developmental abnormalities. We bred CCR5 KO mice with BALB/cJ mice to obtain CCR5 heterozygous mice. Male and female CCR5 heterozygous mice were bred to generate CCR5 KO mice and WT littermates. Genotypes of mice were confirmed by polymerase chain reaction. The sequences of the primers were as follows: forward: 5′-CTACTCCCTGGTATTCATC-3′, reverse: 5′-GGCCTGGTCTAGTCTATT-3′. Female CCR5 KO mice and WT littermates aged 6–8 weeks were used for the study. Mice were housed in a specific pathogen-free environment. MHV-3 was purchased from the American Type Culture Collection, and the MHV-3-FHF model was established according to our previous study [21]. In the pharmacological inhibition of CCR5 experiment, mice received maraviroc (1.23 mg per 20 g mouse weight) by oral gavage daily. All animal protocols were approved by the Tongji Hospital of Tongji Medical School Committees on Animal Experimentation.

### Cell isolation and quantitation

The mononuclear cells (MNCs) in the liver and peripheral organs, including the blood, spleen and bone marrow (BM), were collected as previously described [22].

### Purification of NK cells

An NK Cell Isolation Kit II (Miltenyi Biotec, Germany) was used to purify NK cells according to the manufacturer’s standard protocol. The sorted cells were > 90% pure. The cell viability was > 95%.

### Isolation of hepatocytes

Hepatocytes were isolated from fresh liver tissue by a two-step hepatic portal vein perfusion technique as previously described [8].

### Real-time PCR assays

TRIzol reagent (Invitrogen, USA) was used to extract total RNA from hepatic NK cells, hepatocytes and liver tissue at 0, 24, 48, and 72 h after MHV-3 infection according to the manufacturer’s standard protocol. Subsequently, the total RNA was reverse transcribed into cDNA using a ReverTra Ace qPCR RT kit (TOYOBO, Japan). Real-time quantitative PCRs were performed using SYBR Green Real-time PCR Master Mix (TOYOBO, Japan). The sequences of the primers used are listed in Additional file 1: Table S1.

### Histology and immunohistochemistry

Fresh liver tissues acquired from mice at 0, 24, 48 and 72 h post MHV-3 infection were fixed, embedded and sectioned (4 μm). After haematoxylin and eosin staining, the percentage of necrosis was evaluated by ImageJ software. To detect the expression of MIP-1α, MIP-1β and RANTES, the sections were stained with the following antibodies: MIP-1α (R&D Systems, USA), MIP-1β (R&D Systems, USA), and RANTES (R&D Systems, USA). The sections were observed under a microscope (CX22, OLYMPUS, Japan).

### Immunofluorescence

Tissue paraffin sections were dewaxed and subjected to antigen retrieval treatment. The sections were incubated with the following antibodies: anti-mouse CD3 (Abcam, USA), anti-mouse CD49b (Abcam, USA) and anti-mouse CCR5 (eBioscience, USA). The secondary antibodies were HRP-conjugated goat anti-rabbit (SeraCare, USA). Then, the sections were incubated with FITC-Tyramide and Cy3-Tyramide. DAPI (Bioqiandu, China) was used for nuclear staining. The sections were observed under a fluorescence microscope (BX53, OLYMPUS, Japan). The CD3 + cells were considered T cells, and the CD3-CD49b + CCR5 + cells were considered CCR5 + cNK cells.

### Gene array

Hepatic NK cells of normal BALB/cJ mice and MHV-3-infected BALB/cJ mice (48 h post infection) were isolated and purified, total RNA was extracted, and a gene array was constructed. The Mouse Genome 430 2.0 Array (Affymetrix, USA) was used according to the manufacturer’s standard protocol.

### Liver cytokine measurement

Fresh liver tissues were homogenized on ice in 1 mL of tissue protein extraction reagent (Boster, China) that contained 1% protease inhibitor cocktail (Boster, China). Samples were vortexed, thawed on ice, and centrifuged. The supernatants were collected to analyse MIP-1α (Boster, China), MIP-1β (R&D systems, USA), and RANTES (Boster, China) levels by ELISA, in accordance with the manufacturer’s protocol.

### Transwell migration assay

Transwell migration assays were performed using 6.5 mm Transwell inserts with a 5 µm pore size (Corning, USA) within individual wells of 24-well ultra-low attachment plates (Corning, USA). The wells of the 24-well ultra-low attachment plates were individually filled with 0.6 mL (6 × 10^6^ cells/well) of normal hepatocyte suspension, 0.6 mL (6 × 10^6^ cells/well) of MHV-3-infected hepatocyte (24 h post MHV-3 infection) suspension, 0.6 mL (6 × 10^6^ cells/well) of MHV-3-infected hepatocyte (24 h post MHV-3 infection) suspension with anti-MIP-1α/MIP-1β/RANTES neutralizing antibodies (10 μg/mL, 6 µg/mL, 20 µg/mL, respectively, all from R&D systems, USA), and 0.6 mL RPMI 1640 medium. Rat IgG1 Isotype Control (R&D systems, USA) and Rat IgG2A Isotype Control (R&D systems, USA) were used as isotype controls. Normal NK cells and MHV-3-infected NK cells (24 h post MHV-3 infection) isolated from the spleen with or without anti-CCR5 neutralizing antibodies (BD, USA) were added in 100 µL (1 × 10^5^ cells/well) into the upper chamber, in contact with the medium in the wells. Purified anti-mouse CD191 (CCR1) antibody (Biolegend, USA) and rat IgG2c kappa isotype control (Novus, USA) were used as controls. Then, the plates were incubated for 8 h. NK cells were collected from the bottom chambers after 8 h of incubation. The density of cells was evaluated by a cell counter. Total cells were calculated via density and volume. A FACS assay was used to confirm the proportion of NK cells, and then we calculated the total number of migrated NK cells.

### Flow cytometry analysis

Cells were preincubated with Mouse BD Fc Block (clone 2.4G2, BD Biosciences) before staining. To identify rNK and cNK cells, cells were incubated with antibodies against surface markers: APC-Cy7-conjugated anti-mouse CD3 antibody (clone 17A2), PerCP-Cy5.5-conjugated anti-mouse NKP46 antibody (clone 29A1.4), APC-conjugated anti-mouse CD49a antibody (clone HMα1), FITC-conjugated anti-mouse CD49b antibody (clone HMα2), and PE-conjugated anti-mouse CCR5 antibody (clone HEK/1/85a). APC/Cy7-conjugated Rat IgG2b, κ Isotype Ctrl Antibody (clone RTK4530), PerCP/Cy5.5-conjugated Rat IgG2a, κ Isotype Ctrl Antibody (clone RTK2758), APC-conjugated Armenian Hamster IgG Isotype Ctrl Antibody (clone HTK888), FITC-conjugated Armenian Hamster IgG Isotype Ctrl Antibody (clone HTK888), PE-conjugated Rat IgG2a, κ Isotype Ctrl Antibody (clone RTK2758). All antibodies were purchased from BioLegend (California, USA). Cells were finally subjected to flow cytometry with a BD LSR II flow cytometer (BD Biosciences). The CD3-NKP46 + cells were considered NK cells. Within the CD3-NKP46 + gate, the CD49a + CD49b− cells were considered rNK cells, and the CD49a-CD49b + CCR5 + cells were considered CCR5 + cNK cells.

### Adoptive NK cell transfer

Isolated and purified splenic NK cells were resuspended in PBS, and 5 × 10^4^ cells were intravenously injected into CCR5 KO mice at 0 h, 24 h, and 48 h post MHV-3 infection.

### Statistical analysis

Data gained from experiments are shown as the mean ± SD. To compare differences between multiple groups, one-way ANOVA with post hoc Bonferroni correction (GraphPad Prism 4.02; GraphPad Software) was employed. Group differences in cell migration and liver damage were analysed using two-way ANOVA, with the factors being treatment and genotype, followed by Bonferroni post hoc correction (GraphPad Prism 4.02; GraphPad Software). The log-rank test was used to analyse the survival rate (GraphPad Prism 4.02; GraphPad Software). Statistical significance was identified when* P* < 0.05.

## Results

### CCR5 was highly expressed in hepatic NK cells in the MHV-3-FHF model

**Fig. 1:**
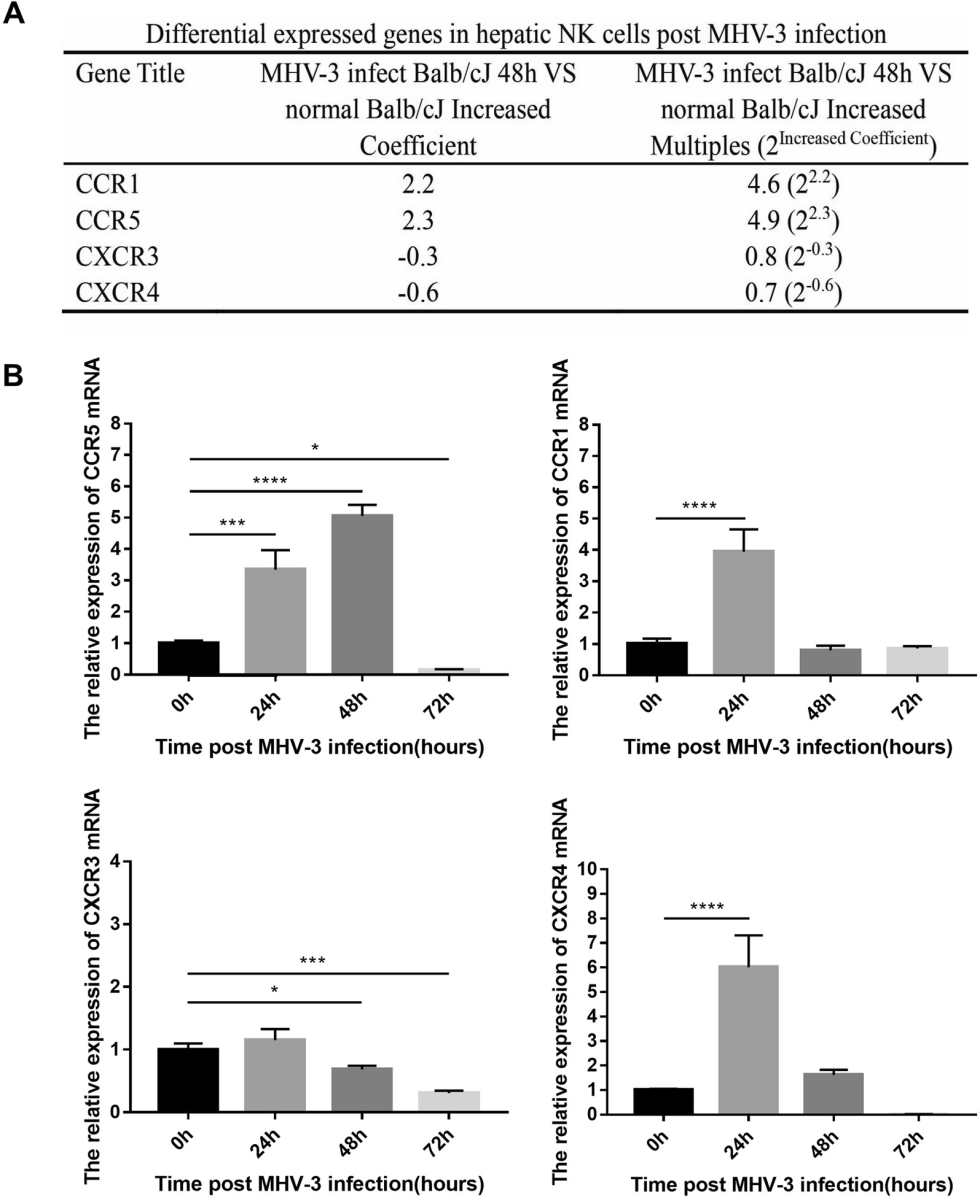
CCR5 was highly expressed in hepatic NK cells in WT mice post MHV-3 infection. **A** The gene chip technique was used to filter out chemokine receptors CCR1, CCR5, CXCR3 and CXCR4 located at the surface of NK cells in the liver with differences in expression between MHV-3-infected and uninfected WT mice. **B** Real-time quantitative PCR was used to investigate the mRNA expression levels of the chemokine receptors CCR1, CCR5, CXCR3, and CXCR4 on the surface of hepatic NK cells at 0, 24, 48 and 72 h post MHV-3 infection. All data are presented as the mean ± SD (n ≥ 6). *P < 0.05 , ***P < 0.001, and ****P < 0.0001

To screen the distinct expression levels of chemokine receptors in NK cells in the liver, a gene array was conducted before and after MHV-3 infection. The expression levels of chemokine receptors CCR1, CCR5, CXCR3 (C-X-C motif chemokine receptor 3) and CXCR4 were significantly changed (Fig. 1A). The different expression levels of chemokine receptors and chemokines are shown in Additional file 1: Table S2. To verify the results, real-time quantitative PCR techniques were applied to investigate the mRNA expression levels of the chemokine receptors CCR1, CCR5, CXCR3, and CXCR4 in hepatic NK cells at 0 (baseline), 24, 48, and 72 h post infection. The results indicated that the mRNA expression levels of CCR5 gradually increased and peaked at 48 h and were restored to baseline levels at 72 h. However, the expression levels of CCR1 and CXCR4 markedly increased and peaked at 24 h and were restored at 48 h. However, CXCR3 expression did not change significantly (Fig. 1B).

### The number of hepatic CCR5^+^ conventional NK cells was significantly elevated post MHV-3 infection

**Fig. 2:**
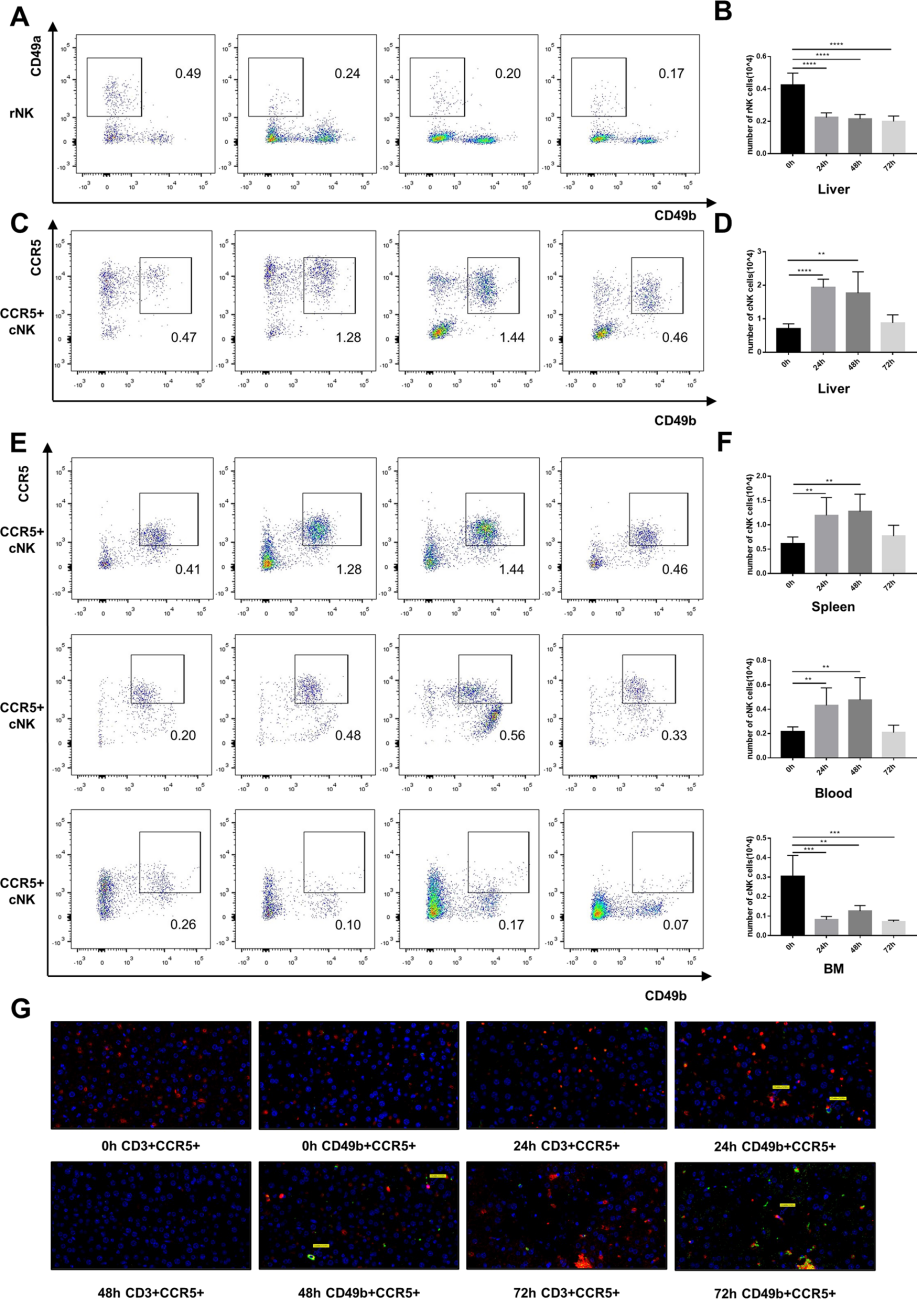
Significant hepatic accumulation of CCR5 + cNK cells in WT mice post MHV-3 infection. **A** Representative flow cytometric plots showing rNK cells in the liver. **B** Number of rNK cells in the liver at 0, 24, 48 and 72 h post MHV-3 infection. **C** Representative flow cytometric plots showing CCR5+ cNK cells in the liver. **D** Number of CCR5+ cNK cells in the liver at 0, 24, 48 and 72 h post MHV-3 infection. **E** Representative flow cytometry plots showing CCR5 + cNK cells in the spleen, peripheral blood and bone marrow. **F** Number of CCR5 + cNK cells in the spleen, peripheral blood and bone marrow at 0, 24, 48 and 72 h post MHV-3 infection. **G** Double immunofluorescence staining showing the infiltration of CD49b + (green)CCR5 + (red) cNK cells and CD3 + (green)CCR5 + (red) T cells in liver tissue at 0, 24, 48 and 72 h post MHV-3 infection (400 ×). All data are presented as the mean ± SD (n ≥ 6). **P < 0.01, ***P < 0.001, and ****P < 0.0001

To evaluate the role of CCR5 in cNK or rNK migration to the liver, we counted the number of rNK and CCR5^+^ cNK cells at 0, 24, 48 and 72 h post infection by flow cytometry. After MHV-3 infection, the number of rNK cells in the liver consistently declined (Fig. 2A, B). However, the number of CCR5^+^ cNK cells in the liver significantly increased and peaked at 48 h (Fig. 2C, D). The percentage of CCR5 + cNK cells in total NK cells increased from 22.78 ± 3.725% (baseline) to 33.2 ± 2.842% (48 h post MHV-3 infection). The percentage of CCR5− cNK cells in total NK cells increased from 0.57 ± 0.373% (baseline) to 0.99 ± 0.356% (48 h post MHV-3 infection). The percentage of CCR5 + rNK cells in total NK cells decreased from 23.90 ± 3.672% (baseline) to 3.08 ± 0.507% (48 h post MHV-3 infection). The percentage of CCR5− rNK cells in total NK cells decreased from 0.93 ± 1.379% (baseline) to 0.27 ± 0.233% (48 h post MHV-3 infection). The number of CCR5^+^ cNK cells in the spleen and peripheral blood showed the same tendency. However, the number of CCR5^+^ cNK cells in bone marrow significantly declined post infection (Fig. 2E, F). The double immunofluorescence staining experiment showed that the infiltration of CCR5^+^ cNK cells began to significantly increase at 24 h post MHV-3 infection, and that the number of migrated CCR5^+^ cNK cells was higher than that of migrated T cells (Fig. 2G).

### Expression of CCR5 ligands was dramatically elevated post MHV-3 infection

**Fig. 3:**
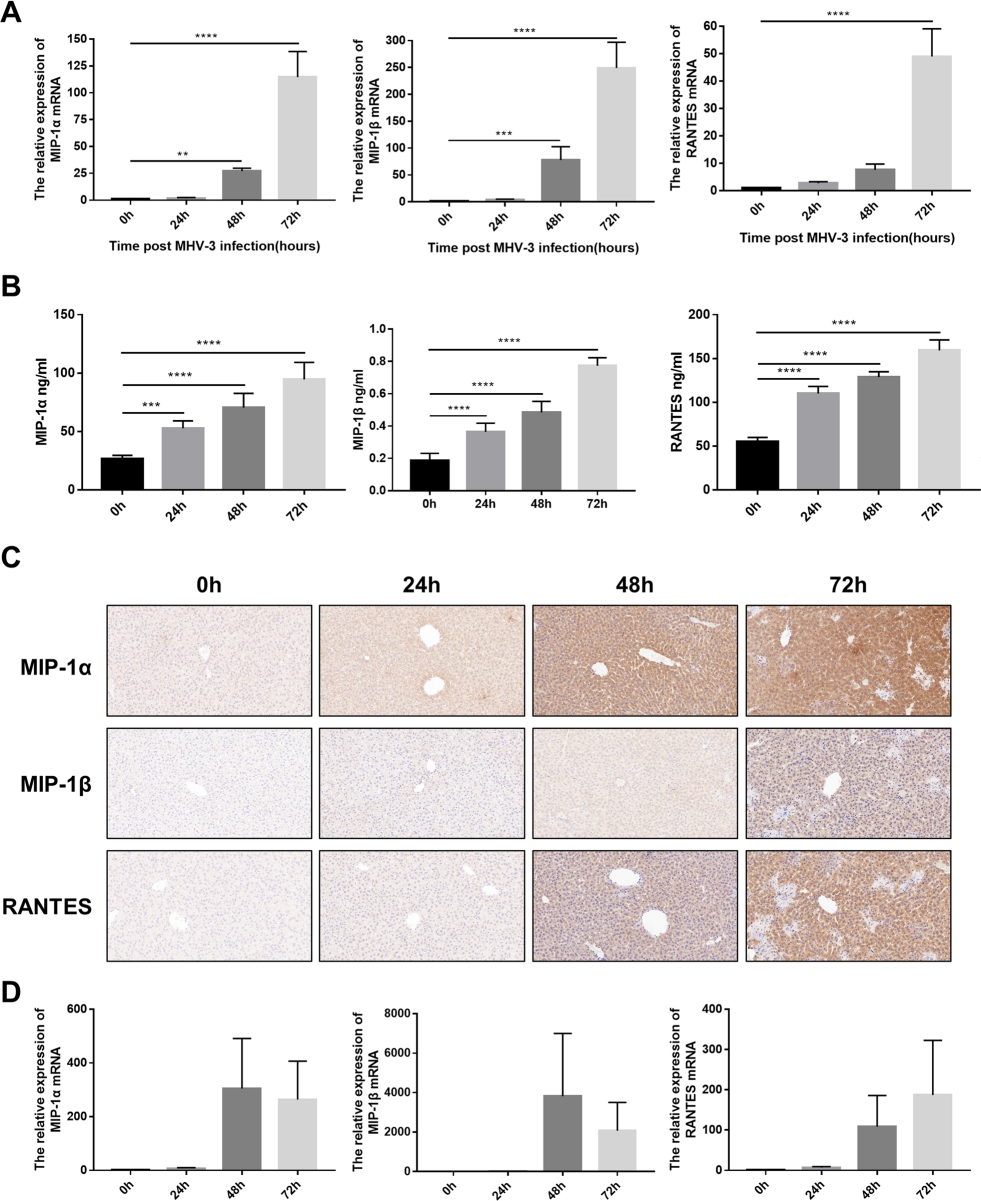
Hepatic chemokines MIP-1α, MIP-1β and RANTES were significantly upregulated in WT mice post MHV-3 infection. **A** Real-time quantitative PCR showing the mRNA expression levels of the CCR5-correlated chemokines MIP-1a, MIP-1β, and RANTES in liver tissues at 0, 24, 48, and 72 h post MHV-3 infection. **B** ELISA showing the expression levels of the CCR5-corresponding chemokines MIP-1α, MIP-1β and RANTES in liver tissues at 0, 24, 48, and 72 h post MHV-3 infection. **C** Immunohistochemical staining showing the expression of MIP-1α, MIP-1β and RANTES in liver tissue at 0, 24, 48, and 72 h post MHV-3 infection. (400 ×). **D** Real-time quantitative PCR measuring the mRNA expression levels of CCR5-corresponding chemokines MIP-1a, MIP-1β, and RANTES in hepatocytes at 0, 24, 48, and 72 h post MHV-3 infection. All data are presented as the mean ± SD (n ≥ 6). **P < 0.01, ***P < 0.001, and ****P < 0.0001

To identify the CCR5 chemotaxis axis, we measured the hepatic expression of the CCR5 ligands MIP-1α, MIP-1β and RANTES at 0, 24, 48 and 72 h post MHV-3 infection. The mRNA levels of CCR5 ligands showed significantly upregulated expression over time (Fig. 3A). Consistently, the hepatic protein level of CCR5 ligands markedly increased from baseline to 72 h post MHV-3 infection according to ELISA and immunohistochemistry detection (Fig. 3B, C). Moreover, the expression of these ligands in hepatocytes was also significantly upregulated (Fig. 3D).

### Blocking CCR5, MIP-1β and RANTES individually decreased cNK cell migration in vitro

**Fig. 4:**
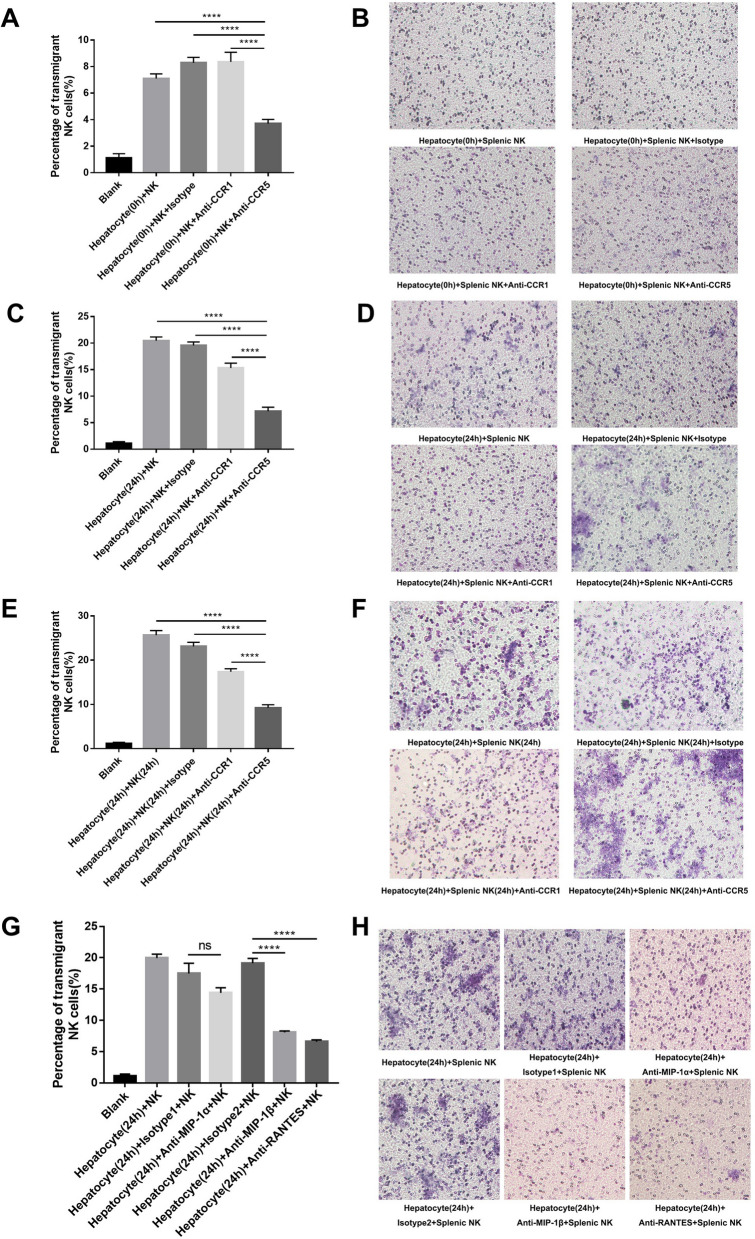
Individual blockade of CCR5 or the ligands MIP-1β and RANTES decreased cNK cell migration to MHV-3-infected hepatocytes in vitro. **A** Percentage of splenic NK cells that transmigrated to normal hepatocytes with isotype control, CCR1 blockage and CCR5 blockage. **B** Transwell migration assay showing the splenic NK cell migration to normal hepatocytes with isotype control, CCR1 blockage and CCR5 blockage. **C** Percentage of splenic NK cells transmigrated to MHV-3-infected hepatocytes with isotype control, CCR1 blockage and CCR5 blockage. **D** Transwell migration assay showing the splenic NK cell migration to MHV-3-infected hepatocytes with isotype control, CCR1 blockage and CCR5 blockage. **E** Percentage of MHV-3-infected splenic NK cells transmigrated to MHV-3-infected hepatocytes with isotype control, CCR1 blockage and CCR5 blockage. **F** Transwell migration assay showing the MHV-3-infected splenic NK cell migration to MHV-3-infected hepatocytes with isotype control, CCR1 blockage and CCR5 blockage. **G** Percentage of splenic NK cells that transmigrated to MHV-3-infected hepatocytes with isotype control and CCR5-corresponding chemokine (MIP-1α, MIP-1β and RANTES) blockage. **H** Transwell migration assay showing the splenic NK cell migration to the MHV-3-infected hepatocytes with isotype control and CCR5-corresponding chemokine (MIP-1α, MIP-1β and RANTES) blockage. All data are presented as the mean ± SD (n ≥ 6). ****P < 0.0001

To determine the exact chemotaxis pathway in cNK accumulation in the liver, a Transwell assay was conducted. Normal hepatocytes (hepatocyte 0 h) recruited a small number of normal splenic NK cells (splenic NK), which could be blocked by an anti-CCR5 antibody instead of an anti-CCR1 antibody (Fig. 4A, B). Moreover, Transwell assays between MHV-3-infected hepatocytes (hepatocytes 24 h) and normal splenic cNK cells (splenic NK) (Fig. 4, C and D) and between MHV-3-infected hepatocytes (hepatocytes 24 h) and MHV-3-infected splenic cNK cells (splenic NK 24 h) (Fig. 4E, F) showed similar tendencies: MHV-3-infected hepatocytes showed strong recruitment of splenic cNK cells and MHV-3-infected splenic cNK cells, which could be largely blocked by an anti-CCR5 antibody. Although the anti-CCR1 antibody could also partly block the process of recruitment, its blocking effects were inferior to those of the anti-CCR5 antibody. Compared with the isotype control, pretreatment with anti-MIP-1β or anti-RANTES antibody, rather than anti-MIP-1α antibody significantly decreased the ability of MHV-3-infected hepatocytes to recruit splenic cNK cells (Fig. 4G, H).

### MHV-3-infected CCR5 KO mice showed less hepatic cNK cell accumulation and attenuated liver injury

**Fig. 5:**
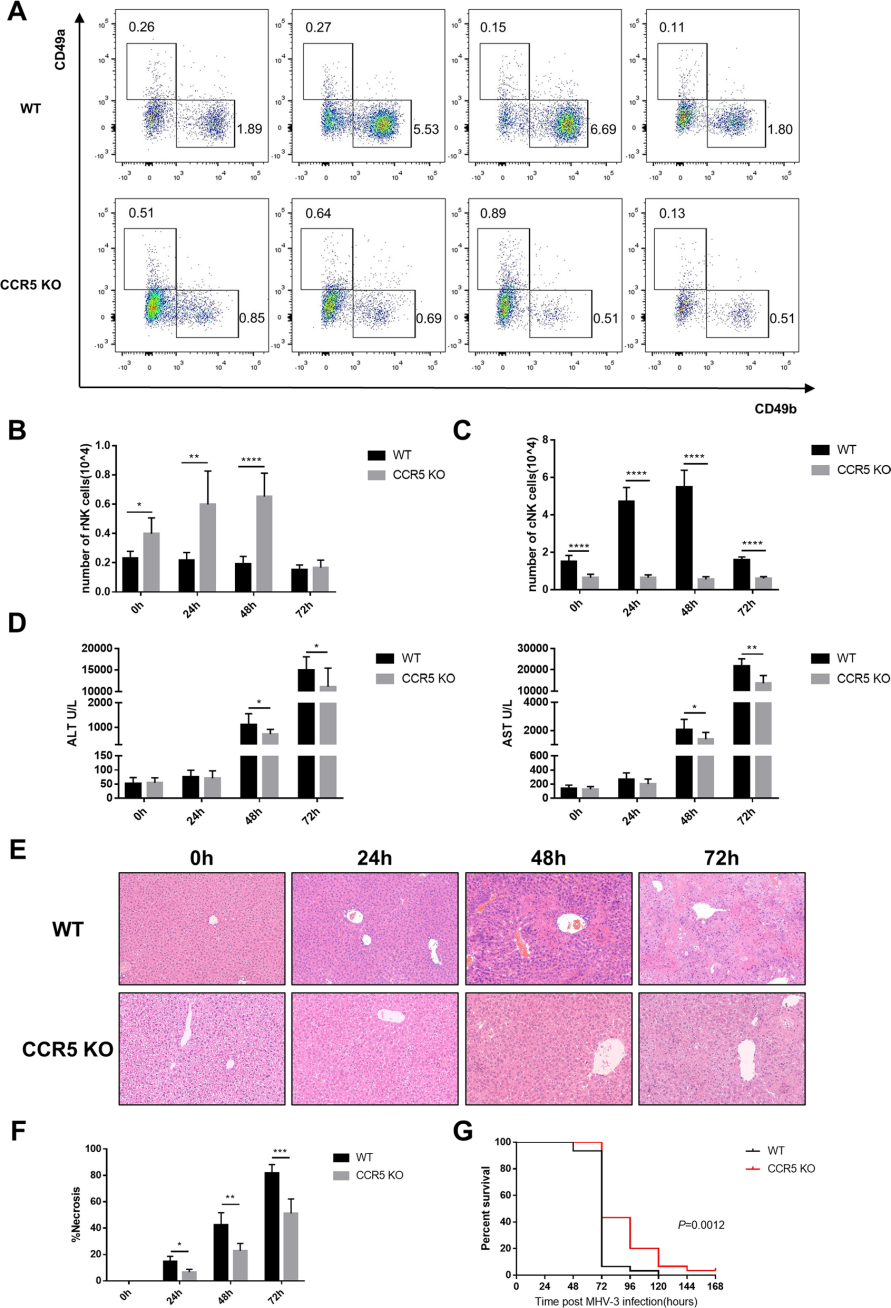
CCR5 knockout mice displayed reduced infiltration of cNK cells and attenuated liver injury post MHV-3 infection. **A** Representative flow cytometric plots showing hepatic rNK and cNK cells at 0, 24, 48 and 72 h post MHV-3 infection in WT and CCR5 KO mice. **B** Number of hepatic rNK cells at 0, 24, 48 and 72 h post MHV-3 infection in WT and CCR5 KO mice. **C** Number of hepatic cNK cells at 0, 24, 48 and 72 h post MHV-3 infection in WT and CCR5 KO mice. **D** Quantification of serum ALT and AST levels. **E** Liver histology (H&E staining, 200 ×) showing necrotic areas and infiltrating cells. **F** Quantification of the necrotic area fraction. **G** Survival curve of WT and CCR5 KO mice post MHV-3 infection. All data are presented as the mean ± SD (n ≥ 6). *P < 0.05, **P < 0.01, ***P < 0.001, and ****P < 0.0001

To further evaluate the role of CCR5 in cNK accumulation in the liver, CCR5 KO mice were infected with MHV-3, and the number of hepatic cNK and rNK cells and the degree of liver injury were determined. Interestingly, the number of hepatic rNK cells in CCR5 KO mice was significantly higher than that in wild-type mice at 0, 24 and 48 h after MHV-3 infection (Fig. 5B). In contrast, the number of hepatic cNK cells in CCR5 KO mice was invariable during MHV-3 infection and was significantly lower than that in wild-type mice at 0, 24, 48 and 72 h post MHV-3 infection (Fig. 5C). As shown in Fig. 5D, E, F, CCR5 KO mice exhibited ameliorated liver damage, as proven by lower serum transaminase levels and a reduced necrosis area fraction in the liver tissue at 48 and 72 h after infection. In addition, the survival time of CCR5 KO mice was obviously improved compared with that of wild-type mice (Fig. 5G).

### Adoptive transfer of wild-type cNK cells aggravated liver injury in CCR5 KO mice

**Fig. 6:**
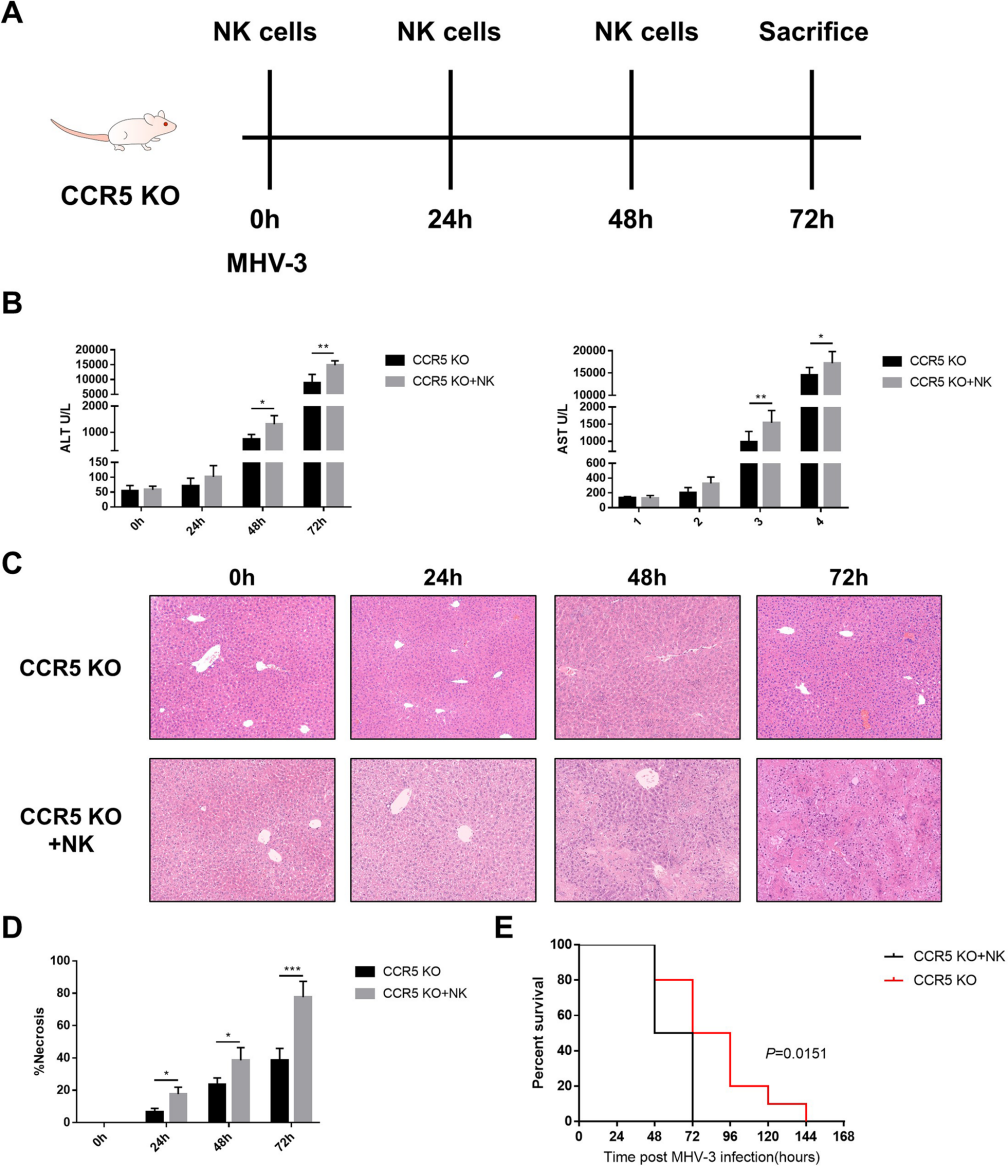
Conventional NK cells migrating to the liver aggravated liver injury in MHV-3-induced fulminant hepatic failure model mice. **A** Adoptive transfer of splenic cNK cells to CCR5 KO mice at 0, 24 and 48 h post MHV-3 infection. **B** Quantification of serum ALT and AST levels. **C** Liver histology (H&E staining, 200 ×) showing necrotic areas and infiltrating cells. **D** Quantification of the necrotic area fraction. **E** Survival curve of control and adoptive transfer groups of CCR5 KO mice post MHV-3 infection. All data are presented as the mean ± SD (n ≥ 6). *P < 0.05, **P < 0.01 and ***P < 0.001

To evaluate liver damage during the accumulation of CCR5^+^ cells in the liver, CCR5 KO mice were adoptive transferred with splenic cNK cells from wild-type mice (Fig. 6A). After the transfer, much more severe liver damage was observed, as evidenced by higher serum transaminase levels (Fig. 6B), an expanded necrotic fraction (Fig. 6C, D) and reduced survival time (Fig. 6E).

### The CCR5 inhibitor maraviroc reduced cNK cell hepatic infiltration and liver injury

**Fig. 7:**
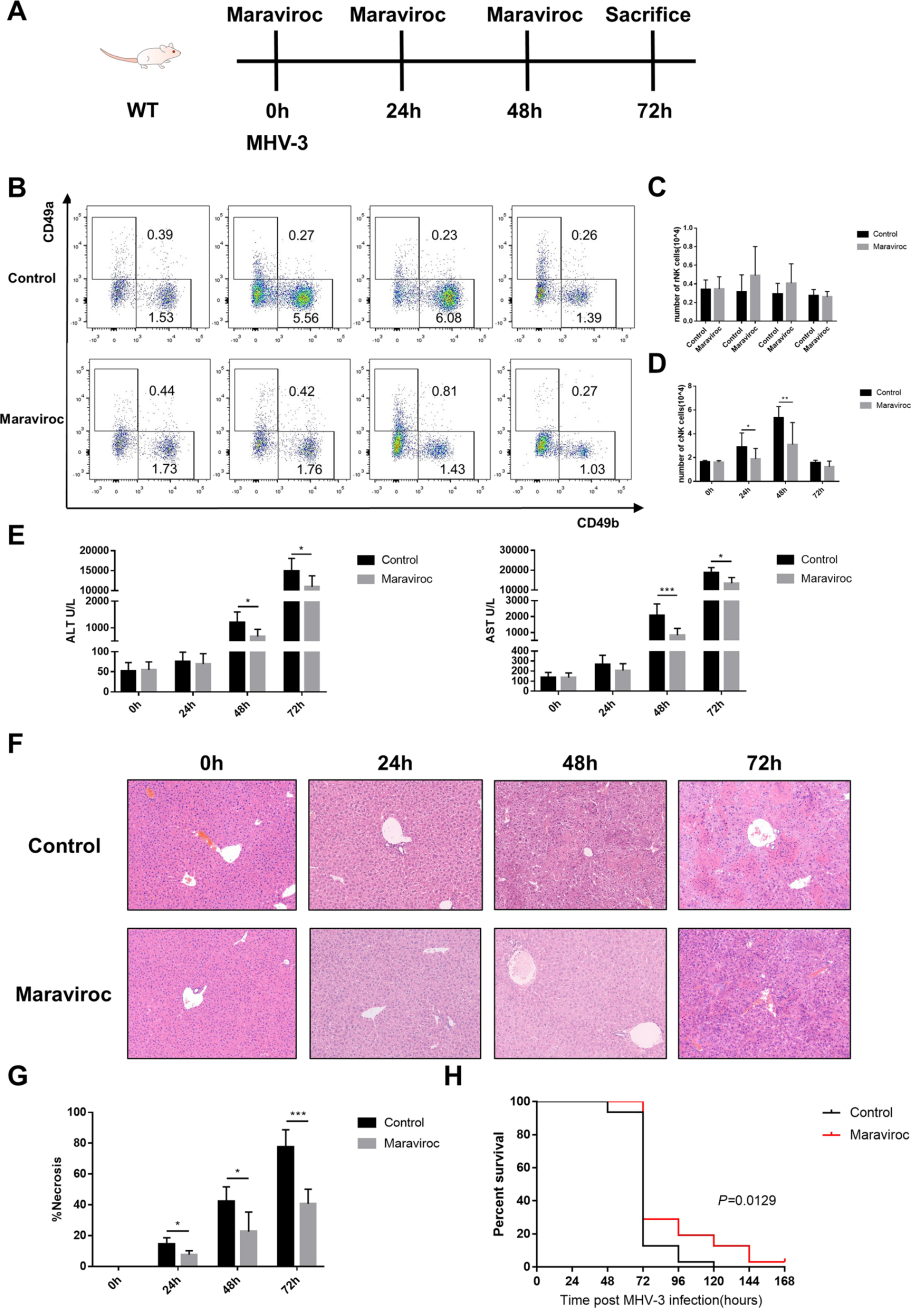
Pharmacological inhibition of CCR5 blocked hepatic cNK cell infiltration and ameliorated liver injury post MHV-3 infection. **A** WT mice directly received the CCR5 inhibitor maraviroc by oral gavage at 0, 24 and 48 h post MHV-3 infection. **B** Representative flow cytometric plots showing hepatic rNK and cNK cells at 0, 24, 48 and 72 h post MHV-3 infection in WT mice with or without maraviroc treatment. **C** Number of hepatic rNK cells at 0, 24, 48 and 72 h post MHV-3 infection in WT mice with and without maraviroc treatment. **D** Number of hepatic cNK cells at 0, 24, 48 and 72 h post MHV-3 infection in WT mice with and without maraviroc treatment. **E** Quantification of serum ALT and AST levels. **F** Liver histology (H&E staining, 200 ×) showing necrotic areas and infiltrating cells. **G** Quantification of the necrotic area fraction. **H** Survival curve of the control and maraviroc groups of WT mice post MHV-3 infection. All data are presented as the mean ± SD (n ≥ 6). *P < 0.05 **P < 0.01, and ***P < 0.001

To assess the therapeutic effect of the CCR5 inhibitor in virus-induced FHF, maraviroc (a CCR5 antagonist) was administered to the MHV-3-FHF mouse model (Fig. 7A). We found no significant difference in the number of hepatic rNK cells between the maraviroc treatment group and the control group during MHV-3 infection (Fig. 7C). However, maraviroc-treated mice displayed a significant inhibition of cNK cell accumulation in the liver during MHV-3 infection (Fig. 7D) and significantly attenuated liver damage, as determined by reduced serum transaminase levels (Fig. 7E), a lower necrotic fraction (Fig. 7F, G) and improved survival time (Fig. 7H).

## Discussion

Acute liver failure is mainly caused by hepadnavirus infection in developing countries and is characterized by rapid progression and high mortality [1, 2]. The host immune system plays crucial roles in the pathogenic mechanism of liver injury, in which the innate immune system has been shown to be an important effector.

Emerging evidence has revealed that NK cells accumulate in the liver and play crucial roles in liver damage [21, 23]. Our previous study showed that enhanced hepatic NK cell death by NKG2D/NKG2D ligand and Fas/Fas ligand contributed to hepatocyte necrosis in an MHV-3-FHF mouse model, and depletion of NK cells at 24 h post MHV-3 infection via anti-ASGM-1 could improve survival in an MHV-3-FHF model [8]. Our current study further elucidated that peripheral cNK cells might play an important role in acute liver injury via recruitment to the liver after MHV-3 infection, which indicated that NK cell chemotaxis might be a potential treatment target for the disease [8].

Chemokines have been proven to be crucial in NK cell migration and chemotaxis. Chemokines are a family of small, basic, heparin-binding proteins with molecular weights of 8–14 kD that play important roles in the recirculation and recruitment of different leukocyte subsets in response to inflammatory stimulation [24–26]. NK cells have been reported to express numerous chemokine receptors, including CXCR1-CXCR4, CCR4, CCR5, CCR7, CCR8 and CX3CR1 (C-X3-C motif chemokine receptor 1) [27–30]. However, their individual roles in NK cell chemotaxis in vivo have not been fully elucidated. Although the chemokine MIP-1α has been reported to be necessary for early NK cell migration into the liver in a cytomegalovirus infection model of mice [31], the specific MIP-1α interacting receptor on NK cells has not been identified. In a *Toxoplasma gondii* infection model, CCR5 KO mice were found to be more susceptible to infection by the pathogen, which might be related to a reduced immune-mediated tissue injury. These previous studies suggested that CCR5 was essential for NK cell migration into the infected liver or spleen [32].

Our study revealed the differential expression of chemokine receptors in hepatic NK cells post MHV-3 infection by microarray. CCR5 and CCR1 were found to be highly expressed in NK cells, and further quantitative PCR verified the results. The results of quantitative PCR showed that the expression level of CCR5 in NK cells increased and peaked at 48 h, whereas CCR1 levels increased only from 0 to 24 h and declined to baseline at 48 h post MHV-3 infection. These results suggested that CCR5, as a prominent receptor, guides NK cell trafficking into the MHV-3-infected liver. The number of CCR5^+^ cNK cells in the BM constantly declined after MHV-3 infection, which was confirmed by flow cytometry. In addition, the number of CCR5^+^ cNK cells in the liver increased and peaked at 48 h and then fell back at 72 h post MHV-3 infection. These results suggested that CCR5 + cNK cells migrate to the liver from the spleen or BM post MHV-3 infection. Furthermore, there was an early and rapid production of the CCR5-corresponding ligands MIP-1α, MIP-1β and RANTES in the liver that consistently increased after MHV-3 infection, which attracted CCR5 + cNK cells to the liver from the BM. These results were consistent with our previous research: after MHV-3 infection, NK cells migrated to the liver from peripheral organs; hence, the number of hepatic NK cells substantially increased and peaked at 48 h post MHV-3 infection [8]. In an HBV murine infection model, CCR5 KO mice infected with adenovirus containing the overlapping HBV1.3 construct (AdHBV) showed an increase in innate immune cells, especially CD11b + NK cells, which were recruited via the CXCL10-CXCR3 axis [33]. However, The MHV-3-FHF model was more likely to imitate the liver failure process of acute virus infection, while the AdHBV model was more similar to a chronic or persistent infection. The differences of virus strain, mouse strain, processes of diseases and outcomes between virus infection studies might contribute to the biological and immunological differences, especially number or functional dynamic changes of NK sub-populations. The Transwell migration assay verified that MIP-1α. MIP-1β and RANTES were essential for cNK cell chemotaxis in MHV-3-induced FHF. Conclusively, we demonstrated that hepatogenic MIP-1α, MIP-1β and RANTES recruited cNK cells to the liver in a CCR5-dependent manner after MHV-3 infection.

Moreover, we showed that the number of cNK cells migrating to the liver in CCR5 KO mice was substantially reduced compared with that in WT littermates after MHV-3 infection. In addition, CCR5 KO mice presented evidently alleviated liver injury and prolonged survival time. In our study, we innovatively used a CCR5 antagonist in the MHV-3-FHF model. The CCR5 inhibitor maraviroc has already been used to ameliorate the progression of hepatic steatosis in an in vivo model of nonalcoholic fatty liver disease [17]. Strikingly, application of the CCR5 inhibitor maraviroc, when administered directly post MHV-3 infection, resulted in a clear amelioration of liver damage, alongside a marked decline in the infiltration of cNK cells in the liver, indicating that the reduced cNK cell infiltration accounted for the improvement after maraviroc treatment. Adoptive transfer experiments confirmed that cNK migration to the liver was the critical factor in the progression of MHV-3-induced FHF and implied that CCR5 was required for cNK cell trafficking to infected liver cells.

In conclusion, our data suggest that CCR5 and its corresponding ligands MIP-1β and RANTES play crucial roles in the hepatic recruitment of cNK cells in MHV-3-induced and immune-mediated liver injury. Maraviroc, a CCR5 antagonist, might effectively resolve NK cell-mediated liver injury in severe viral hepatitis and liver failure. This study highlighted the profound impact of altered chemokine receptors on the innate immune response in virus-induced liver injury and a potential therapeutic strategy for acute severe liver damage.

### Supplementary Information

**Additional file 1: Table S1. **Primer sequences used for real-time PCR**. Table S2. **Differential expressed genes in hepatic NK cells post MHV-3 infection.

## Data Availability

The datasets generated during and/or analyzed during the current study are available from the corresponding author on reasonable request.

## Abbreviations

AdHBV: Adenovirus containing the overlapping HBV1.3 construct
BM: Bone marrow
CCL-3: Chemokine (C–C motif) ligand 3
CCL-4: Chemokine (C–C motif) ligand 4
CCL-5: Chemokine (C–C motif) ligand 5
CCR5: Chemokine (C–C motif) receptor 5
cNK cells: Conventional NK cells
FHF: Fulminant hepatic failure
GPCR: G-protein-coupled receptor
MHV-3: Murine hepatitis virus strain 3
MIP-1α: Macrophage inflammatory protein 1α
MIP-1β: Macrophage inflammatory protein 1β
MNCs: Mononuclear cells
NK cells: Natural killer cells
RANTES: Regulated on activation, normal T cell expressed and secreted
rNK cells: Resident NK cells
