# Supplementary material for: C–C chemokine receptor 5 is essential for conventional NK cell trafficking and liver injury in a murine hepatitis virus-induced fulminant hepatic failure model
Source: J Transl Med. 2023 Nov 29;21:865. doi: 10.1186/s12967-023-04665-8 (PMC10685630; doi:10.1186/s12967-023-04665-8)
Supplement: Supplementary file 1 — Additional file 1: Table S1. Primer sequences used for real-time PCR. Table S2. Differential expressed genes in hepatic NK cells post MHV-3 infection. [file 12967_2023_4665_MOESM1_ESM.docx]

**Table S1. Primer sequences used for real-time PCR**

| Gene | Sense (5'-3') | Anti-sense (5'-3') |
| --- | --- | --- |
| CCR1 | ACCCAGTGAGAAGAAGGTCA | CCAGGTCCAGTTGCTTACTC |
| CCR5 | CTACTTTCTCTTCTGGACTC | AGGCATAGATGACAGGGTT |
| CXCR3 | GAGAGCAAATGTGGATGTTG | GAGAGCAAATGTGGATGTTG |
| CXCR4  MIP-1α  MIP-1β  RANTES  GAPDH | GTCAACCTCTACAGCAGCGT  CATGACACTCTGCAACCAAGTCTTC  CCATGAAGCTCTGCGTGTCTG  ACCAGCAGCAAGTGCTCCAA  CTCATGACCACAGTCCATGCCATC | GTAAAGGCGGTCACAGATGT  GAGCAAAGGCTGCTGGTTTCA  GGCTTGGAGCAAAGACTGCTG  TGGCTAGGACTAGAGCAAGCAATG  CTGCTTCACCACCTTCTTGATGTC |

**Table S2. Differential expressed genes in hepatic NK cells post MHV-3 infection**

| Gene Title | MHV-3 infect Balb/cJ 48h VS normal Balb/cJ Increased Coefficient | MHV-3 infect Balb/cJ 48h VS normal Balb/cJ Increased Multiples (2^Increased Coefficient^) |
| --- | --- | --- |
| CCR1 | 2.2 | 4.6 (2^2.2^) |
| CCR like 2 | 1.2 | 2.3 (2^1.2^) |
| CCR5 | 2.3 | 4.9 (2^2.3^) |
| CCR9 | -2.6 | 0.2 (2^-2.6^) |
| CX3CR1 | 1.9 | 3.7 (2^1.9^) |
| CXCR3 | -0.3 | 0.8 (2^-0.3^) |
| CXCR4 | -0.6 | 0.7 (2^-0.6^) |
| CCL5 | 0.1 | 1 (2^0.1^) |
| CCL6 | 0.1 | 1 (2^0.1^) |
| CCL9 | -0.4 | 0.8 (2^-0.4^) |
| CCL12 | 5.8 | 55.7 (2^5.8^) |
| CXCL2 | 1 | 2 (2^1^) |
| CXCL10 | 4.1 | 17.1 (2^4.1^) |
| CXCL16 | -3.5 | 0.1 (2^-3.5^) |
